# Adapting practice in mental healthcare settings during the COVID-19 pandemic and other contagions: systematic review

**DOI:** 10.1192/bjo.2021.20

**Published:** 2021-02-26

**Authors:** Jessica Raphael, Rachel Winter, Katherine Berry

**Affiliations:** Research and Innovation, Greater Manchester Mental Health NHS Foundation Trust, UK; and Division of Psychology and Mental Health, School of Health Sciences, Faculty of Biology, Medicine and Health, Manchester Academic Health Sciences, University of Manchester, UK; Research and Innovation, Greater Manchester Mental Health NHS Foundation Trust, UK; and Division of Psychology and Mental Health, School of Health Sciences, Faculty of Biology, Medicine and Health, Manchester Academic Health Sciences, University of Manchester, UK; Division of Psychology and Mental Health, School of Health Sciences, Faculty of Biology, Medicine and Health, Manchester Academic Health Sciences, University of Manchester, UK

**Keywords:** COVID-19, service changes, mental health services, clinical governance, best practice

## Abstract

**Background:**

During the global COVID-19 pandemic, there has been guidance concerning adaptations that physical healthcare services can implement to aid containment, but there is relatively little guidance for how mental healthcare services should adapt service provision to better support staff and patients, and minimise contagion spread.

**Aims:**

This systematic review explores service adaptations in mental health services during the COVID-19 pandemic and other contagions.

**Method:**

The Allied and Complementary Medicine database (AMED), the Cumulative Index to Nursing and Allied Health Literature (CINAHL), EMBASE, Medline, PsycINFO and Web of Science were systematically searched for published studies from database inception to April 2020. Data were extracted focusing on changes to mental health services during contagion outbreaks. Data were analysed with thematic analysis.

**Results:**

Nineteen papers were included: six correspondence/point-of-view papers, five research papers, five reflection papers, two healthcare guideline documents and one government document. Analysis highlighted four main areas for mental health services to consider during contagion outbreaks: infection control measures to minimise contagion spread, including procedural and practical solutions across different mental health settings; service delivery, including service changes, operational planning and continuity of care; staff well-being (psychological and practical support); and information and communication.

**Conclusions:**

Mental health services need to consider infection control measures and implement service changes to support continuity of care, and patient and staff well-being. Services also need to ensure they are communicating important information in a clear and accessible manner with their staff and patients, regarding service delivery, contagion symptoms, government guidelines and well-being.

In December 2019, Wuhan, a city in China, reported an outbreak of a novel coronavirus, later named SARS-CoV-2, or COVID-19. Its global spread caused countries to take varying measures to contain the virus, to differing effect.^1^ Equally, health services had to quickly adapt to minimise risk of contamination of staff and patients. Recent guidance provided by the World Health Organization^2^ highlighted changes that physical healthcare services can implement to maintain service delivery and mobilise the workforce. There is also growing literature emphasising the importance of service changes to support staff well-being during contagions,^3,4^ and the provision of additional mental health services as a response to increased mental health difficulties in the general public.^5–7^ However, there is little guidance how mental healthcare services should adapt service provision to better support staff and patients and maintain service delivery despite a number of challenges within these settings, such as the effect of isolation on increasing mental health symptoms,^8,9^ an increase in the number of patients needing to access in-patient settings,^10^ increased patient vulnerability (particularly when faced with social circumstances such as unemployment,^11^ which has increased as a result of the COVID-19 pandemic) and poor service provisions owing to reduced funding.^12^ Additionally, patient lack of capacity could affect patient ability to adhere to new guidelines around contagion containment,^13^ such as social distancing. Because of the relative lack of literature on mental health service adaptations during the COVID-19 pandemic, this work was undertaken to systematically review and synthesise literature regarding adaptations to practice in mental health settings during global and localised (non-global)/viral or infectious disease outbreaks. By exploring current and historical recommendations relating to COVID-19 and other airborne, human-to-human contact and blood and body fluid contagions such as Ebola, SARS and HIV outbreaks, which also prove fatal for humans, we aim to provide a guide for good practice and service adaptations across mental health services to minimise risks and better support mental health workers and patients during global and localised contagion.

## Method

### Systematic review

#### Search strategy

A review protocol was registered (International Prospective Register of Systematic Reviews registration number: CRD42020186969) before starting data searching and extraction. The review adopted the Preferred Reporting Items for Systematic Reviews and Meta-Analyses (PRISMA) guidelines.^14^ Searches were undertaken using the Allied and Complementary Medicine database (AMED), the Cumulative Index to Nursing and Allied Health Literature (CINAHL), EMBASE, Medline, PsycINFO and Web of Science. Databases were searched from inception to 30 April 2020, using search terms listed in Table 1. Searches were limited to English-language publications (or those translated to English). Additional studies were identified by scanning the references of those included papers.

**Table 1:** Search strategy

1. Population	2. Situation	3. Intervention
Mental illness* mental disorder* mentally ill mental difficult* psychiatr* psychol*	Pandemic global cris* Local* infect* SARS Severe acute respiratory syndrome Spanish flu* MERS Middle East Respiratory Syndrome Contagion contagious virus* COVID-19 Corona* Epidemic* Ebola	Adapt* chang* address* meet* recommend* guideline* action* polic* practic* practis*
Search: 1 using OR Search: 2 using OR Search: 3 using OR Search: 4 using OR Search: 1 & 2 & 3 & 4 Search fields: title, abstract and keywords (following abstract)		

#### Eligibility criteria

Inclusion criteria were peer-reviewed articles (qualitative and quantitative studies, literature reviews, briefings, reflective/discussions based on author experience and commentaries/point-of-view based on author expertise), federal government guidelines, official international guidelines and World Health Organization recommendations; reference to mental health services/staff/patients (including out-patient and in-patient services); and reference to any adaptations to practice during any global pandemic, epidemics or localised infections (non-global contagions)/outbreaks related to viruses and infectious diseases.

Exclusion criteria were books/book chapters, conference abstracts and newspaper articles; sole reference to physical health services, mental health in the general population and mental health needs in physical health staff; sole reference to any virus or diseases that do not result in a pandemic, epidemic or localised infection; no mention of adaptations to practice; and description of provision of mental health services for people with infectious diseases (e.g. HIV) rather than service adaptations in response to an outbreak.

#### Study selection

All studies meeting the eligibility criteria were imported into a referencing software program (Endnote version 9 for Windows) and duplicate references identified and deleted. Initially, the first author independently screened titles and abstracts identified from the systematic search for relevance, using the inclusion criteria set out above. The second author independently screened 50% of the titles and abstracts of the literature. Where both authors agreed on exclusions, titles and abstracts were discarded. Where both authors agreed on inclusion the full-text article was retrieved. Disagreements were resolved through discussion between both authors. Following discussion, both authors agreed either the paper did or did not meet criteria, when uncertainty remained, full texts were retrieved. Moderate levels of agreement were found between the raters (*κ* = 0.583) at the title and abstract stage. In the second stage, both authors independently assessed 100% of the full texts against the inclusion criteria. Where both authors agreed on exclusion, full texts were discarded and reasons for exclusion noted. Almost perfect agreement were found between the raters (*κ* = 0.975). Disagreements regarding inclusion were discussed by the first and second author, and where disputes were not resolved, a third reviewer arbitrated.

#### Assessing rigour and credibility of included studies

To enable the reader to judge rigour, eligible papers were described using the Critical Appraisal Tool to Assess the Quality of Cross-Sectional Studies (AXIS) (Supplementary Table 1).^15^ The tool was selected because of the different study designs and methodologies the review incorporated. The authors agreed on definitions of the quality appraisal tool questions (see Supplementary Table 2 available at https://doi.org/10.1192/bjo.2021.20). The first stage involved the first and second author independently assessing the quality of the papers. The second stage involved checking the authors’ level of agreement; the authors had complete agreement.

#### Data extraction and analysis

Data were extracted using a data extraction tool developed in Microsoft Excel version 16 for Windows by the authors, which required the authors to extract author, year, country, article type, method, sample, results and changes to service. Data were extracted from each paper from the relevant sections of the included papers: recommendations within commentaries and guidelines, plus methods, results and discussion sections. The first author checked 100% of the data extracts extracted by the second author. and the second author checked 54% of the data extracts of the first author. There were no disagreements with the data extracted. Data were analysed using thematic analysis informed by Braun and Clarke,^16^ because of the range of different studies included in the systematic review and a lack of prior theory. First, the first and second author familiarised themselves with the data to systematically assess the findings from each study, understand similarities and difference between papers and highlight important paper characteristics. The data were coded initially by using an inductive, data-driven approach, by the first author. When exploring relationships within and between papers, data saturation was achieved after ten papers. Codes that appeared related were grouped into ‘code families’ by the first author. The first author wrote a codebook including a brief description about each code family. The second author then applied the codebook to the remaining papers. Following completion of initial coding, the codebook was refined through discussion between the first and second author. After the codebook was refined, the first and second author dual-coded each other's coding. There were no conflicts. The first author reviewed code families for similarities and generated themes; themes were then discussed between the first and second author, who collaboratively refined, defined and named the themes.

## Results

### Overview of studies and quality appraisal

**Fig. 1:**
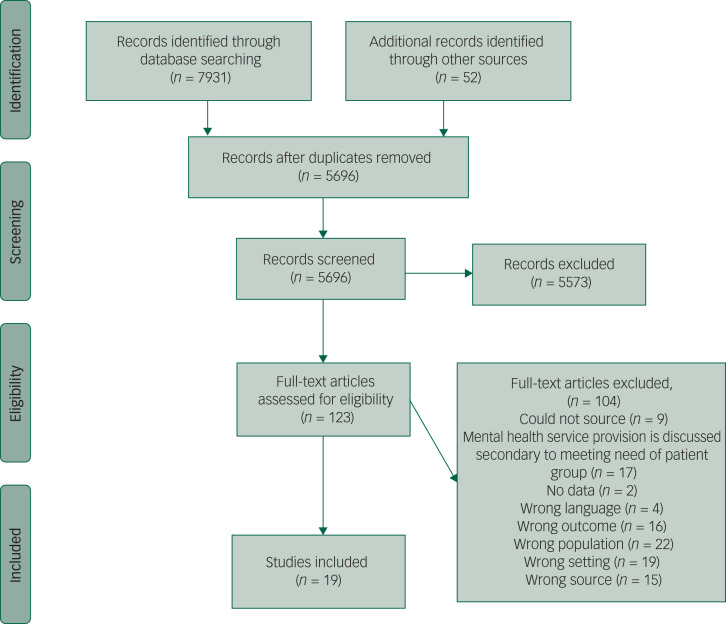
Preferred Reporting Items for Systematic Reviews and Meta-Analyses diagram.

Nineteen papers met inclusion criteria (Fig. 1, Table 2). There were six correspondence/point-of-view papers,^21,26,31,33,35^ five research papers,^17,23,27,29,30^ five reflection papers,^18–20,22,25^ two healthcare guidelines^24,34^ and one government document.^32^ Five papers were from the USA,^19–22,33^ three from Canada,^17,30,32^ two were global,^24,27^ two from France^18,23^ two from Italy,^31,34^ two from China,^28,35^ one from Sierra Leone,^25^ one from Taiwan^26^ and one from Australia.^28^ Papers covered a wide range of contagions, including COVID-19,^18,21,26–28,31,33–35^ HIV,^19,20^ influenza,^22,23,29,32^ non-specified pandemics,^17,24^ Ebola^25^ and non-specified infectious diseases.^30^ Results of the quality appraisal tool are reported in Supplementary Table 1. AXIS^15^ scores show that the included papers were of moderate to good quality. Based on the included studies, the majority were considered appropriate study/report design for the aim (*n* = 14), and the discussions and conclusions justified by the results (*n* = 12). However, it is important to highlight that the majority of included papers did not use a specific research design as defined by Babbie,^36^ O'Sullivan et al^37^ or Creswell.^38^ No studies were omitted from analysis.^39^

**Table 2:** Overview of studies

Author, year	Country	Study type	Sample (including number, where applicable)	Type of contagion	Method
Amaratunga et al (2007)^17^	Canada	Peer-reviewed research paper	Three hospital pandemic plans from three Ontario cities	General pandemic	Qualitative gap analysis of three hospital pandemic plans, using a checklist of 11 support categories: aggregate plans for hospitals within own geographical regions (two hospital plans included mental health hospitals)
Chevance et al (2020)^18^	France	Reflection paper	Not applicable	COVID-19	Narrative review identifying relevant guidelines to delivering mental healthcare during the COVID-19 pandemic by reviewing results in scientific and medical literature and in local initiatives in France
Cournos et al (1989)^19^	USA	Reflection paper	One state hospital	HIV	Discussion of problems faced by state hospitals in New York in managing patients with HIV infection and how management approaches have evolved over 5 years
Cournos et al (1990)^20^	USA	Reflection paper	Five case studies of HIV in two State hospitals in New York	HIV	Case study
Druss (2020)^21^	USA	Correspondence/point of view	Not applicable	COVID-19	Not applicable
Duley (2005)^22^	USA	Reflection paper	Healthcare system in Connecticut, including 31 acute care hospitals, the Veteran's Administration Hospital in West Haven, Hospital for Special Care, Gaylord Rehabilitation Hospital, Natchaug Psychiatric Hospital and the State's 13 community health centres	Influenza	Discussion of planning activities for pandemic influenza through observations, personal experiences and governmental guidance's, reports and plans
Gaspard et al (2014)^23^	France	Peer-reviewed research paper	19 units: 17 hospital units (four geriatric psychiatry, 13 clinical psychiatry), two residential homes (one specialised and one medical)	Influenza	Develop monitoring in a psychiatric hospital, to improve knowledge and validate the alert and control measures in such settings. Each year, teams made aware of flu risks. If flu was noticed in some units, alert messages were sent to all other units
Inter-Agency Standing Committee (2007)^24^	Global	Healthcare guidelines	Not applicable	General pandemic	Guidelines to enable the planning, establishment and coordination of a set of minimum multi-sectoral responses to protect and improve people's mental health and psychosocial well- being in the middle of an emergency
Kamara et al (2017)^25^	Sierra Leone	Reflection paper	One support service	Ebola	Description of experience establishing a nurse-led mental health and psychosocial support service in a 300-bed hospital
Kim and Su (2020)^26^	Taiwan	Correspondence/point of view	Not applicable	COVID-19	Correspondence regarding seriousness of COVID-19 in those with serious mental illness
Liebrenz et al (2020)^27^	Global	Peer-reviewed research paper	Not applicable	COVID-19	Literature review followed by recommendations
Liu et al (2020)^28^	China	Correspondence/point of view	Not applicable	COVID-19	Correspondence of challenges to deliver mental health services during COVID-19 pandemic
Maguire et al (2009)^29^	Australia	Peer-reviewed research paper	*N* = 309 (71 with confirmed schizophrenia recruited from community and hospital settings and 238 attending a general practitioner without this diagnosis); population aged 18–65 years	Influenza	Questionnaire related to swine influenza pandemic, exploring risk perception and willingness to undertake protective measures (measured by self-rated health question), K10 was also used to measure anxiety and depression subscales
Musau et al (2015)^30^	Canada	Peer-reviewed research paper	Selective sampling: bedside nurses and nurse managers from acute care hospital, including those from surgical, medical, nephrology and psychiatric units; *N* = 28 participants for individual interviews	General infectious disease	Retrospective exploratory case study design: secondary data analysis of infection rates, interviews and document analysis
Percudani et al (2020)^31^	Italy	Correspondence/point of view	Not applicable	COVID-19	Short communication with recommendations for occupational health and safety of patients and staff
Public Health Agency of Canada (2006)^32^	Canada	Government document	Not applicable	Influenza	Policy document drawing on dialogues within the Pandemic Influenza Committee, and those with wider stakeholders (health non-government organisations, local governments, emergency planners and bioethicists)
Ripp et al (2020)^33^	USA	Correspondence/point of view	Mount Sinai Health System	COVID-19	Rapid needs assessment model to assess the concerns of their workforce conducted by a task force at MSHS
Starace and Ferrara (2020)^34^	Italy	Healthcare guidelines	Not applicable	COVID-19	Actions, proposed by the Italian Society of Epidemiological Psychiatry, to Italian Mental Health Departments during the COVID-19 pandemic
Zhu et al (2020)^35^	China	Correspondence/point of view	Not applicable	COVID-19	Letter to Editor addressing risk and preventative measures for catching COVID-19 on a psychiatric in-patient ward

K10, 10-item Kessler Psychological Distress Scale; MSHS, Mount Sinai Health System.

### Synthesis

Four main themes were developed: infection control measures to minimise contagion spread, service delivery, staff well-being and information and communication. Each theme will be discussed as a set of recommendations for mental health services during localised and global contagion outbreaks. A checklist of mental health services changes has been provided (Table 3), with supporting quotes from relevant papers.

**Table 3:** Service change checklist

Theme	Service actions	Supporting quote
Infection control	All settings:	
	Staff symptom monitoring	‘They are required to self-monitor for the appearance of symptoms on a daily basis’ (Chevance et al, page 5)^18^
	Patient symptom monitoring	‘Monitoring for respiratory and other Covid-19 symptoms, including temperature, was carried out daily’ (Percudani et al, page 2)^31^
	Hand hygiene protocols	‘The hospital placed hand sanitizers in and outside all patient rooms, beside patient beds and at regular intervals in the hallways throughout the hospital’ (Musau et al, page 5)^30^
	Prescription protocol review	‘With the decree of March 15th, pharmacies are authorized to accept expired prescriptions in cases of chronic illness and treatment prescribed for at least 3 months, until May31st to limit interruptions in treatment’ (Chevance et al, page 6)^18^
	Social distancing	‘In the waiting area, social distancing must be guaranteed (therefore, the number of people allowed should be carefully monitored, chairs can be moved/removed from the area)’ (Starace and Ferrara, page 2)^34^ ‘During this outbreak, social distancing or wearing masks might help us to prevent infection, yet don't let these measures prevent us from expressing compassion and friendliness’ (Kim and Su, page 2)^26^
	Alternative transportation for staff to get to work (in place of public transport)	‘Transportation has also become a challenge as public transit and shared rides put health care workers (and the people traveling with them) at risk, but single passenger options are financially unsustainable. Our institution has started to offer staff free parking and bike rental options as well as to make arrangements for reduced-cost car rentals’ (Ripp et al, page 2)^33^
	Out-patient settings:	
	Open-air home visits	‘If possible, home visit should be performed in open air. Minimal interpersonal distance should be maintained’ (Starace and Ferrara, page 3)^34^
	In-patient settings:	
	Review of admission policies and alternative services	‘The hospitals would implement more stringent triage and decrease length of hospital stay. There also would be an attempt to decrease other types of admissions if possible…Admission criteria should be reviewed and revised as necessary’ (Duley, page 353)^22^
	Patient health checks	‘For new admissions, check the physical health status (specifically, the presence of cough, body temperature >99.5 F, sore throat, shortness of breath), and contacts at risk in the previous 14 days’ (Starace and Ferrara, page 3)^34^
	Reduce opportunities for contagions to get onto wards: stop leave and visits	‘The work within Residential Psychiatric Facilities continued as normal although external activities were suspended, including home leave and temporary permissions and visits of relatives’ (Percudani et al, page 2)^31^
	Reduce opportunities for contagions to get onto wards: implement staff dressing area	‘In this area, a filter room was set up for dressing and undressing’ (Percudani et al, page 3)^31^
	Reduce opportunities for contagions to get onto wards: use only specific providers	‘In principle, only food and clothing from government-approved institutions are acceptable’ (Zhu et al, page 301)^35^
	Reconfigure wards to include an infected patient area	‘The creation of an integrated COVID + medical/psychiatric system within the medical units, which have been reorganized, thus offering the most appropriate medical and psychiatric care for patients’ (Chevance et al, page 5)^18^
	Reduce group activities	‘Group activities, both those for users and those for family members, are suspended. As an alternative, individual therapy sessions or family meetings can be provided, if necessary’ (Starace and Ferrara, page 3)^34^
	Review cleaning protocols	‘Guidelines for the cleaning and decontamination of equipment and physical plant were also enacted’ (Musau et al, page 4)^30^
Service delivery: service changes	All settings:	
	Good IT infrastructure	‘Services should implement an adequate telemedicine software both on telephones and laptops/computers accessible to all professionals’ (Starace and Ferrara, page 3)^34^
	Out-patient settings:	
	Remote triaging	‘For new and emergent cases, triage is done over the telephone to understand the urgency of care, and when required, an appointment is scheduled’ (Percudani et al, page 2)^31^
	Remote (telephone or video call) services where possible	‘The consultation centres and day care facilities that had to close to comply with health instructions are organizing nursing and medical remote consultations, while maintaining the possibility of face-to-face reception for the most risk-prone situations’ (Chevance et al, page 6)^18^
	Focus on prevention of mental health distress	‘Patients will need support in maintaining healthy habits, including diet and physical activity, as well as self-management of chronic mental and physical health conditions’ (Druss, page 1)^21^
	In-patient settings:	
	Video calls to replace family visits	‘Visits were prohibited and replaced by video conferences’ (Chevance et al, page 5)^18^
	Remote video call meetings for staff	‘Meetings are suspended. When necessary (e.g. multidisciplinary meeting involving different services for a vulnerable situation-discharge from hospital), meetings can be performed through telemedicine tools (such as video call)’ (Starace and Ferrara, page 3)^34^
	Good discharge planning	‘Intensive telephone follow-up should be offered in the days and weeks following hospital discharge in order to prevent the risk of suicide, limit the risk of care interruption and relapse’ (Chevance et al, page 6)^18^
	Proactive patient well-being support: self-care resources, psychoeducation and coping mechanisms	‘While promoting the least unfavourable experience of confinement via adapted psycho-educational tools (information leaflets, telephone evaluation of the confinement experience, support for caregivers)’ (Chevance et al, page 6)^18^
Service delivery: operational planning	All settings:	
	Stockpile resources: PPE, medical equipment, medication	‘Establishing stockpiles of standard infection control supplies (hand hygiene supplies, gowns, gloves, and surgical masks)’ (Duley, page 356)^22^
	Staff resource planning	‘Discuss with community leaders the responsibilities of the community in providing a supportive and protective network. The following groups may be mobilised: Health professionals and, if possible, mental health professionals; When appropriate, local non-allopathic health care providers (e.g. religious leaders, traditional healers: see Action Sheet 6.4); Social workers and other community-based mechanisms (e.g. women's groups, mental health consumer organisations); Family members.’ (Inter-Agency Standing Committee, page 133)^24^
Service delivery: continuity of care	All settings:	
	Maintain scheduled appointments	‘The scheduled appointment should be maintained in the following scenarios: (a) critical clinical situation, as assessed during previous visits or the phone check-in mentioned in point 1, and reported by the patient or caregivers (e.g. current exacerbation of symptoms, manifestation of new side effects, lack of adherence to the pharmacological treatment); (b) the necessity to administer pharmacological therapy at the centre (e.g. long-acting medications, direct observed therapy); (c) legal obligations (mandated to care)’ (Starace and Ferrara, page 2)^34^
	Delivery of psychological therapy through a variety of media	‘Online psychological self-help intervention systems, including online cognitive behavioural therapy for depression, anxiety, and insomnia (e.g. on WeChat), have also been developed’ (Liu et al, page 1)^28^ ‘When suspended, the lead provider organises an alternative therapeutic programme (individual in person or phone/video sessions)’ (Starace and Ferrara, page 3)^34^
	Integrated mental health physical healthcare	‘We have concluded that resources are best used by providing integrated care’ (Cournos et al, page 155)^19^ ‘Enable basic health and mental health care throughout the emergency’ (Inter-Agency Standing Committee, page 135)^24^
	Strong leadership and multi-agency partnerships and defined responsibilities	‘Strong leadership and partnerships between the health ministry and mental health nurses, nongovernmental organizations and hospital management were essential for establishing a successful service’ (Kamara et al, page 844)^25^
Staff well-being	All settings:	
	Provide staff with psychological support (e.g. counselling sessions)	‘Support from colleagues will be essential for maintaining physical, mental, and social well-being, particularly if the pandemic is of an extended duration’ (Druss, page 1)^21^ ‘Emotional support/grief counselling (aimed at permitting workers to continue to work and reduce loss of staff due to grief or traumatic stress)’ (Public Health Agency of Canada, page 360)^32^
	Provide staff with physical support (e.g. access to child care)	‘Child and elder care should be made available for mental health clinicians working extra shifts’ (Druss, page 1)^21^ ‘Basic personal support – ensure food and services are available to HCWs on the job…Family care (for children, seniors, sick family members who do not require hospitalization). This poses significant infection control concerns if gathering children or the elderly together for group care’ (Public Health Agency of Canada, page 360)^32^
Information, communication and training	All settings:	
	Communication needs to be clear and honest	‘It is essential for each hospital and community health centres to communicate honestly and openly with its staff regarding pandemic influenza and the plan the facility has in place or is working on to deal with a pandemic, should it ever come. Health care organizations should communicate directly not only with their staff, but with the population they primarily serve’ (Duley, page 357)^22^
	Communication through a range of mediums	‘Consolidating system-wide messaging into a daily communiqué with links to a comprehensive website has helped streamline messaging and direct our workforce, situated within multiple hospitals and numerous practice sites, to a single regularly updated resource. Weekly system-wide virtual town halls have also helped with delivering essential information’ (Ripp et al, page 2)^33^
	Information for staff and patients on well-being	‘Psychiatrists have a crucial role in informing patients about…measures to prevent and combat the stress linked to the pandemic itself’ (Chevance et al, page 6)^18^ ‘Ensure ongoing, close supervision of those mobilised to provide basic care and provide access to information on how to maintain their own emotional health’ (Inter-Agency Standing Committee, page 133).^24^
	Information for staff and patients on service changes	‘There are four main messages they need to communicate over and over…(2) When is it actually necessary to come to the facility for care related to influenza; (3) Appropriate sites for outpatient triage and care; and (4) Options for self-care’ (Duley, page 357)^22^
	Information for staff and patients on the contagion	‘Communicating information about national and local epidemiology was justified’ (Gaspard et al, page 50)^23^ ‘To further facilitate the exchange of key information regarding infectious diseases, staff huddles are held twice daily during outbreaks’ (Musau et al, page 5) ^30^
	Information for staff and patients on infection control	‘Psychiatrists have a crucial role in informing patients about confinement and barrier measures to limit the spread of the epidemic’ (Chevance et al, page 6)^18^
	Staff training in symptom detection	‘Mental health clinicians need training to recognize the signs and symptoms of this illness and develop knowledge about basic strategies to mitigate the spread of disease for both in their patients and themselves’ (Druss, page 1)^21^
	Staff training in psychological ‘first aid’ for patients	‘To strengthen the skills of Connaught hospitals non-specialist nurses, mental health awareness training was provided by the mental health nurse and King's Sierra Leone Partnership volunteer. A half-day session on psychological first aid’ (Kamara et al, page 843)^25^
	Staff training in PPE	‘Address the need for regular training, and practice drills, for PPE’ (Amaratunga et al, page 206)^17^

PPE, personal protective equipment; HCW, healthcare worker.

#### Infection control measures to minimise contagion spread

Procedural and practical solutions were identified within the data that aimed to minimise spread in either community or in-patient mental health settings.

##### Procedural solutions: all settings

The data described procedural solutions that mental health services could employ to reduce contagion spread. These included monitoring staff and patient symptoms, such as checking patient and staff temperature, testing for contagions, tracking and tracing the contagion, administering vaccinations where possible,^18,19,23,24,31^ and staff reporting their contagion status on a daily basis.^18^

##### Procedural solutions: out-patient settings

To avoid spread across different settings, the data suggested that services adopt other procedural solutions, including reviewing regulations regarding repeat prescription dates so patients are not required to attend face-to-face medication appointments.^18,35^ Where the contagion involves an airborne virus, services can employ social distancing measures,^26,34^ alternative means for staff to get to work avoiding public transport,^30^ and patient home visits in open-air areas.^34^

##### Procedural solutions: in-patient settings

The synthesis found in-patient-specific considerations to minimise infection rates within wards, including reviewing admission criteria policies to reduce the number of patients requiring admission to hospital; reducing admission duration; managing surge capacity;^22,34^ and for services to explore using other care settings within the same radius of acute hospital settings and community services, to meet patient needs.^19,22^ Where admission is still required, mental health in-patient wards should quarantine patients on admission,^17,18^ and conduct physical health checks to establish whether the patient has the contagion.^31,34^ In-patient wards should endeavour to provide patients with single bedrooms,^32,33^ and ask them to stay in their rooms where possible.^23^

Where these options are not feasible, the data suggested that in-patient wards could reduce transmission by stopping leave and visitors,^18,30,31,34^ ensuring food and clothing is only provided through approved channels,^18,30^ reducing staffing where possible^18^ and erecting staff dressing areas for staff to change their clothes before entering and leaving the ward.^18,31^ To control contagions on the ward, services can reconfigure wards to have specific areas for infected patients.^18^ Similarly, group activities can be stopped or reduced in frequency, to minimise spread on the wards.^34^ Infected patients could be placed on continuous observations where it is not possible for them to be confined to a particular area. To protect staff from contracting the contagion, in-patient services should suspend multidisciplinary team meetings or conduct meetings via video calls,^34^ have appointments with patients in outside spaces, and focus on least-restrictive practice with patients by using verbal de-escalation rather than restraint.^19,20^ Where wards use restraint, they should use restraint blankets, retractable needles and increase personal protective equipment (PPE).

##### Practical solutions: all settings

Practical considerations found within the data included regular handwashing for staff, patients and visitors; access to hand-cleaning products;^21,32,34^ and use of PPE for both staff and patients.^17,21,27,30,31,33^ Services referenced staff PPE as essential to protect staff from contracting and spreading the contagion.

##### Practical solutions: in-patient settings

The literature highlighted where PPE were limited, mental health in-patient wards can implement a policy to use PPE for patients on a needs basis. Priorities included patients who had symptoms,^34^ were at high risk of severe symptoms because of underlying health conditions,^23^ lacked capacity^19^ to adhere to alternative measures to minimise spread or were unable to isolate in bedrooms.^30^ Measures to improve cleaning practices during contagions should be used, such as ensuring the ward is decluttered, improving ventilation, assigning patients their own equipment, using more vigorous cleaning controls for equipment and bedding, using disposable crockery and adopting stricter waste management measures.^18,21,32,34^

#### Service delivery

Within the service delivery considerations highlighted in the literature, there are three subthemes: service delivery changes requiring service planning both during outbreaks and for future provision; operational planning requiring services to be adaptable and flexible, responding to ever-changing circumstances and service and patient needs; and maintaining continuity of care.

##### Service changes

The data suggested that out-patient services adopt telephone triaging to assess patient need.^22,27,31^ Therefore, services can prioritise patient care and collaborate with patients regarding the suitability of continuing with prescheduled appointments face to face or remotely. Additionally, clinicians can assess risk and explore whether more frequent contact is required. Following triage, low-risk patients can continue to access care through telephone or video call consultations with clinicians, whereas high-risk patients can continue to receive home visits.^34^ Other remote service options include telephone hotlines for suicide prevention,^18^ developing reactive software that runs algorithms to predict patients at risk of self-harm^28^ and adopting targeted crisis interventions.^34^ Other mental health out-patient service changes include prioritising preventative measures to reduce patient need during contagion outbreaks.^18,19,23,26,30,34^ For example, services can provide patients with coping strategies^34^ and psychological therapy to support patient well-being.^25,28^ Recommendations included providing free telephone counselling,^26,28,33^ continuing therapy or counselling services remotely, increasing access to one-to-one therapy, providing therapy by community and family doctors,^35^ and creating targeted crisis interventions.^26^ However, these recommendations are dependent on all parties having sufficient information technology software and hardware.

For in-patient services, which have stopped visitations, wards can instead hold patient–family video conferences, provide regular telephone updates to families and provide patients with telephones to contact family and friends.^18,31^ Services could ensure patient discharges include consideration of the physical and psychological impact of a contagion, including follow-up calls by ward staff as part of a suicide prevention plan, and to ensure that rapid in-patient to out-patient care transfers are in place.^18,19,35^ In addition to maintaining usual well-being patient standards, including dignity for vulnerable patients, avoiding discrimination and meeting basic physical needs,^24,25^ the literature identified proactive measures services could adopt to support patient well-being. These measures include the provision of self-care guidance; psychoeducation tools for maintaining well-being during confinement;^18,22,25,33^ and monitoring patient well-being and symptoms through regular follow-up telephone calls,^34^ exploring patient long-term needs and conducting risk assessments at discharge to consider the effects of home circumstances and to prevent addiction risks.^18^

##### Operational planning

The data suggested that mental health services should be proactive and have infection resources in place, in addition to reactive planning in relation to factors such as reduced staff resource. In anticipation of contagions, mental health services should stockpile PPE, medical equipment and medication, including antibiotics.^17,22^ Services should have emergency action plans, with details about alternative sites for surge capacity.^22,24^ Additionally, service change plans that are in their infancy should be brought forward, if beneficial for overcoming service demands during contagion.^25^ In readiness for staff shortages as a result of a contagion, services should mobilise a human resources management team to oversee staff resourcing;^17,32^ this would enable quick contact with staff at short notice. Services should identify high-risk areas with gaps in service owing to high staffing shortages, and redeploy staff to services with increased need.^22,24^ If there are insufficient staff to fill gaps, services can recruit and train licensed volunteers; utilise non-healthcare worker support and family care; redeploy students, newly qualified staff and retired staff; and establish a primary response corps involving immunised survivors in the case of a second wave of contagions.^22,24,25,32,33^

##### Continuity of care

The literature stipulated that services should endeavour to provide consistent mental healthcare. Where services are not already meeting basic patient needs before outbreaks, services should continue to deliver basic care as a priority, rather than focusing on service changes during contagion outbreaks.^25^ The literature identified several ways to achieve continuity by maintaining face-to-face appointments with patients where possible; maintaining scheduled appointments related to critical situations, pharmacology and legal obligations; maintaining delivery of psychological therapy sessions; and ensuring acute care services are available.^18,26,31,34,35^ The literature suggested ensuring continuity of care through liaison with agencies (e.g. social care services) could help to prevent patient relapse.^19,22^ Similarly, the literature identified the importance of integrated care to maintain patient well-being, and suggested ways to ensure integration between mental and physical health services, including establishing a specific ward for ‘infected’ patients so as to meet their physical and psychological needs, providing psychological support during contagion testing and carrying out physical and mental health well-being assessments.^18,19,21,24,27,34,35^ When care plans involve multi-agency resources, it is important for services work in partnership and establish the responsible party.^24,25^ Additionally, training staff in anticipation of contagions should be provided, including how to implement infection control measures such as use of PPE.^17,18^ Training should also include monitoring symptoms, and diagnosis and management of the contagion;^18,21,22^ psychological ‘first aid’ for patients;^25^ restraint and aggression management;^19,24^ and how newly mobilised workforces can maintain self-care.^24^ The literature suggested that training should be delivered by external parties to improve staff adherence.^19^ Finally, to maintain high service delivery standards, strong leadership is important during contagion outbreaks. Strong leadership skills included visibility of leaders within services, agreement with other managers regarding the level of patient support provision, ability to form partnerships across services and with other providers and providing ongoing supervision to all staff.^17,25^ The literature also referenced the need for managers to have access to senior support for ethical decision-making.^18^

#### Staff well-being

The review highlighted the importance of considering practical and psychological well-being of staff, and ways to maintain well-being through increased support and resources.^17,18,21,24,25,27,31,33,35^

##### Psychological support

Psychological support included providing staff with information on maintaining well-being;^24,33^ monitoring staff burnout;^17^ offering psychological therapy or counselling sessions either online or via the telephone, including staff-specific crisis lines;^17,18,24,25,27,32,33^ peer support through hotlines and monthly supervision,^21,25^ and supervision of newly mobilised workforces.^24^

##### Practical support

Practical support included recognition of staff commitment,^17^ compensation through financial support and incentives,^17^ guaranteed job protection where staff are redeployed,^32^ providing in-patient staff with food for infection control,^32^ allowing staff time to rest,^32^ free parking and bike rental to reduce reliance on public transport,^33^ child/dependent care for staff who would be unable to work otherwise,^21,22^ and accommodation if staff need to isolate away from those they live with.^21^

#### Information and communication

Data suggested that mental health organisations need to ensure they provide clear and honest information and communication to staff, patients and family members, relating to service changes in response to outbreaks, including a rationale for changes,^17,22,27,30^ signposting to other services where appropriate, and practical measures in place to minimise contagion spread.^21–23,27^ Mental health services should also review their communication plans in light of localised and global contagion outbreaks to ensure communication with staff, patients and families/carers is honest and open, and minimalize sensationalism and stigma relating to infected individuals.^17,29,32^ Similarly, patient information should be provided through multiple sources and by a range of parties, ensuring it is accessibly written and presented for maximum effect.^17,21,33^ For staff, information can be delivered through staff meetings, weekly bulleted emails and on whiteboards in staff areas.^30,33^ Patients and staff should both have access to visible, accessible signs regarding new procedures.^30^ In in-patient settings where visitations have stopped, families should be provided with information about alternative ways to contact their loved ones, and be kept informed of patient well-being.^18,30,32,35^ Finally, the literature suggested that mental health services should provide patients, family members and staff with information to provide knowledge about the contagion itself and ways to minimise contagion spread, including vaccination administration (where applicable), hand hygiene, PPE and social distancing.^22,23,27,30,35^

## Discussion

Since the COVID-19 outbreak, mental health services have relied on using infection control measures and service adaptations that have been primarily directed at physical healthcare settings.^40^ Given the number of challenges mental health settings face as a result of increased patient vulnerability^11^ and poor service provisions attributable to reduced funding,^12^ this review explored how mental health services should adapt practice and procedures during the COVID-19 pandemic, and in anticipation of future contagion outbreaks. The data suggested that a number of adaptations relating to infection control measures, service delivery, staff well-being and information, communication and training.

### Infection control

Our review found out-patient and in-patient mental health settings should use infection control measures during contagion outbreaks, including staff and patient symptom monitoring and strict handwashing hygiene, both of which are in line with Royal College of Nursing^41^ guidance.

Where infections are highly contagious due to ease of spread via respiratory droplets, such as COVID-19,^42^ the data suggested that services should conduct staff meetings and patient appointments via video calls and that any face-to-face appointments could be carried out in open-air areas such as patient gardens. Using video calls to carry out appointments with patients could prove effective given that, in the UK, nine out of ten people have access to the internet at home.^43^ However, services need to consider the patient group when implementing delivery of online appointments, given that research has found that some patients, in particular those who are elderly, can struggle to use the hardware and software required to have an online appointment.^44^ Additionally, there is disparity in access to technology across mental health clinical services;^45^ for example, variation in the UK National Health Service Trusts between using paper versus electronic patient records.^46^

The review also suggested that services should use social distancing measures to reduce contagion spread. However, there are challenges to social distancing in in-patient settings and office spaces.^45,47^ To overcome this, the data recommended that in-patient wards consider reducing the number of admissions to increase the ability to follow social distancing measures; however, recent consultation work has found that the number of reduced admission are often short-lived.^45^ The data suggested that staff and patients could use PPE where social distancing is not possible. However, this provides new challenges, particularly in acute mental health settings, as patients can lack capacity to comply with measures to minimise spread, as found by a previous literature review exploring issues of non-adherence in those with mental health difficulties.^13^ Additionally, a literature view by Edwards et al, exploring emotion recognition in individuals with schizophrenia, found that individuals diagnosed with schizophrenia find it particularly difficult to engage in meaningful communication when they are unable to view facial expressions, because of prior impaired facial affect recognition.^48^

Our data suggested that other infection control measures in-patient wards may consider, including stopping leave, visits and group activities. However, recent consultation work conducted in the UK found that these measures may have a detrimental effect on patient well-being.^45^ It is also noteworthy that previous literature has demonstrated the therapeutic benefits of leave,^49^ meaningful activities^50^ and social support networks on recovery,^51,52^ and therefore caution should be taken where these are stopped.

If patients or staff on in-patient wards contract a viral contagion, the data suggested improving ward ventilation, which is in line with prior international guidelines recommending that natural ventilation for infection control measures in healthcare settings are most effective.^53^

### Service changes

Our review highlights a number of service changes that out-patient mental health services may consider adopting during a contagion outbreak. Patient triage and psychological therapy via telephone and video calls were recommended, in addition to provision of psychoeducation to prevent increase in patient need; a previous review of psychological therapies found that this can reduce patient relapse when provided to patients with serious mental health difficulties^54^ and their family members.^55^ The data also recommended that mental health services should use digital technology, which is in line with recent literature suggesting that healthcare providers should move toward adopting virtual mental health services to meet demand.^56^

In-patient service changes suggested by our review also included supporting patient contact with family members through video calls in replace of visits, and providing family members with regular telephone updates regarding their loved one's well-being. Previous studies have highlighted the benefits of involving families in in-patient care, such as reduced length of admission and relapse prevention,^57,58^ which highlights the importance of maintaining patient contact with families through technology.

#### Operational planning

A number of considerations relating to operational planning were suggested by the review. Given the infection control measures required to minimise contagion spread, the data suggested that services should be prepared by stockpiling PPE and medical equipment. In addition, services should have surge capacity plans, including identifying suitable services to signpost patients to, and ensuring sufficient staffing numbers by redeploying staff to high-need areas and utilising voluntary, retired and other healthcare professionals. Other literature that aimed to assess healthcare preparedness for emergencies has previously identified gaps in in emergency planning within hospital settings relating to stockpiling sufficient equipment and surge capacity plans.^59^

#### Continuity of care

The data found continuity of care was important to prevent patient relapse by maintaining services where possible, and providing integrated mental and physical healthcare. Prior guidance by the World Health Organization demonstrates that integrating care helps services address treatment gaps in a cost-effective manner.^60^ However, prior literature states that if services adopt an integrated approach, they have to provide staff with additional training regarding how to use infection control measures^45^ and how to support patient physical and mental health.^61^

#### Staff well-being

The review stressed the importance of supporting staff during the contagion outbreaks through recognition schemes and supporting child/dependent care for staff who would be unable to work otherwise. Psychological support included providing staff with information on maintaining well-being and offering online or telephone psychological therapy or counselling sessions. Previous literature has highlighted the responsibility of healthcare managers in creating and maintaining staff health through the development of policy initiatives,^62^ and the importance of staff well-being to ensure the delivery of high-quality care.^63^

#### Information and communication

Finally, in support of prior literature that sets out recommendations for providing patients with healthcare information, our review stressed the importance of providing staff and patients with clear and honest information^64^ through a range of media about service changes, the contagion and infection control measures.

#### Strengths and limitations

We found consistency between our review findings and findings from previous literature.^45^ Despite this, it is important to consider the rigour and credibility of the data on the findings. Although the data included moderate to good levels of reporting, we note that where literature included both mental and physical health settings, it was hard to differentiate between the two within the results and discussion; therefore, the findings may not fully consider challenges that are specific to mental health service delivery.^65^ Additionally, the review included a number of different types of data, the majority of which did not use a research design as defined by Babbie,^36^ O'Sullivan et al^37^ or Creswell.^38^ It is therefore important to note that despite the frequencies of themes that occurred within the included papers, only three papers^23,29,30^ conducted research using participant data via known valid and reliable outcome measures. We are therefore unable to comment on whether use of these recommendations result in improved outcomes for staff and patients in mental healthcare settings. To mitigate these limitations, future systematic reviews may be necessary once research has been conducted assessing the effects of changes to service delivery during the COVID-19 pandemic.

##### Implications

It was evident from our work that although there has been research exploring changes to practices in physical health settings, mental healthcare setting were less considered. This is likely because there is not parity of esteem between mental and physical healthcare despite rhetoric that there should be; in comparison to physical health, mental healthcare is underresourced, undervalued and stigmatised.^12^ Lack of guidance for changes to mental health settings during contagion outbreaks can affect staff and patient physical well-being through lack of infection control measures, and lack of consideration toward the effects on staff and patient mental well-being. Therefore, this literature review began to address this gap, to provide guidance regarding how out-patient and in-patient mental health services can adapt their practices in response to contagion outbreaks.

The literature review findings can therefore be used to inform mental health services regarding ways to mitigate risk of COVID-19 spread within the services, and how best to support mental health staff during the pandemic. However, the findings from this review not only relate to COVID-19, but also can form the basis for changes to practices for other contagions, whether they are local, epidemic or pandemic outbreaks. Additionally, the findings provide recommendations about service adaptions during an outbreak, and inform future emergency planning within mental health services. These recommendations could aid service preparedness for future outbreaks. Finally, given the specific challenges that mental health in-patient wards face,^45^ the findings of the review can help services understand ways to overcome these challenges within the in-patient setting.

In conclusion, we provide findings from the first systematic review of service adaptations in mental health settings during the global COVID-19 pandemic and other more localised contagions. Mental health services need to consider infection control measures and implement service changes to support continuity of care and patient well-being. Mental health services also need to consider the effects of COVID-19 or other localised contagion outbreaks on their staff, and support staff well-being. Throughout contagion outbreaks, mental health services need to ensure that they are communicating important information with their staff and patients regarding service delivery, contagion symptoms, government guidelines and well-being, in an honest and accessible manner.

## Data Availability

Data availability is not applicable to this article as no new data were created or analysed in this study.

## Supplementary material

For supplementary material accompanying this paper visit http://dx.doi.org/10.1192/bjo.2021.20. click here to view supplementary material
