# Supplementary material for: Adapting practice in mental healthcare settings during the COVID-19 pandemic and other contagions: systematic review
Source: BJPsych Open. 2021 Feb 26;7(2):e62. doi: 10.1192/bjo.2021.20 (PMC8027557; doi:10.1192/bjo.2021.20)
Supplement: Supplementary file 1 [file S205647242100020Xsup001.docx]

Supplementary material

Table. 1 Study AXIS scores (Y = Yes, N= No, D/K = Don’t know, and N/A = Not applicable)

| **Question** | Amaratunga et al (2007) | Chevance et al (2020) | CourNs et al (1989) | CourNs et al (1990) | Druss (2020) | Duley (2005) | Gaspard et al (2013) | Inter-Agency Standing Committee (2007) | Kamara et al (2017) | Kim & Su (2020) | Liebrenz et al. (2020) | Liu et al (2020) | Maguire et al (2009) | Musau et al (2015) | Percudani et al (2020) | Public Health Agency of Canada (2006) | Ripp et al (2020) | Starace & Ferrara (2020) | Zhu et al (2020) |
| --- | --- | --- | --- | --- | --- | --- | --- | --- | --- | --- | --- | --- | --- | --- | --- | --- | --- | --- | --- |
| 1. Were the aims/objectives of the report clear? | Y | Y | Y | Y | N | Y | Y | Y | Y | Y | N | N | Y | Y | N | Y | Y | Y | N |
| 2. Was the study/report design appropriate for the stated aim(s)? | Y | Y | Y | Y | N/A | Y | Y | Y | Y | Y | N/A | N/A | Y | Y | N/A | Y | Y | Y | N/A |
| 3. Was the sample size justified? | Y | D/K | D/K | Y | N/A | D/K | D/K | N/A | N/A | N/A | N/A | N/A | N | D/K | N/A | N/A | D/K | N/A | N/A |
| 4. Was the target/reference population clearly defined? (Is it clear who the research was about?) | Y | Y | Y | Y | Y | Y | Y | Y | Y | Y | Y | N | Y | Y | Y | Y | N | Y | Y |
| 5. Was the sample frame taken from an appropriate population base so that it closely represented the target/reference population under investigation? | D/K | Y | Y | Y | N/A | Y | Y | N/A | Y | N/A | N/A | N/A | Y | Y | N/A | N/A | Y | N/A | N/A |
| 6. Was the selection process likely to select subjects/participants/data that were representative of the target/reference population under investigation? | Y | D/K | Y | Y | N/A | Y | Y | N/A | Y | N/A | N/A | N/A | Y | Y | N/A | N/A | D/K | N/A | N/A |
| 7. Were measures undertaken to address and categorise Nn-responders? | N/A | N/A | N/A | N/A | N/A | N/A | N/A | N/A | N/A | N/A | N/A | N/A | D/K | D/K | N/A | N/A | D/K | N/A | N/A |
| 8. Were the outcome variables/data measured appropriate to the aims of the study? | Y | Y | D/K | Y | N/A | Y | Y | N/A | N/A | N/A | N/A | N/A | Y | Y | N/A | Y | Y | N/A | N/A |
| 9. Were the outcome variables/data measured correctly using instruments/measurements that had been trialled, piloted or published previously? | Y | N | N | Y | N/A | D/K | D/K | N/A | N/A | N/A | N/A | N/A | Y | Y | N/A | N/A | D/K | N/A | N/A |
| 10. Is it clear what was used to determine statistical significance and/or precision estimates? (e.g. p-values, confidence intervals) | N/A | N/A | N/A | N/A | N/A | N/A | Y | N/A | N/A | N/A | N/A | N/A | Y | N/A | N/A | N/A | N/A | N/A | N/A |
| 11. Were the methods (including statistical methods) sufficiently described to enable them to be repeated? | Y | N | N | Y | N/A | N | Y | N/A | N | N/A | N/A | N/A | Y | Y | N/A | N | N | N/A | N/A |
| 12. Were the basic data adequately described? | Y | N | N | Y | N/A | N | Y | N/A | Y | N/A | N/A | N/A | Y | Y | N/A | N/A | Y | N/A | N/A |
| 13. Does the response rate raise concerns about Nn-response bias? | D/K | N/A | N/A | N/A | N/A | N/A | N/A | N/A | N/A | N/A | N/A | N/A | D/K | D/K | N/A | N/A | N/A | N/A | N/A |
| 14. If appropriate, was information about Nn-responders described? | N | N/A | N/A | N/A | N/A | N/A | N/A | N/A | N/A | N/A | N/A | N/A | N | N | N/A | N/A | N/A | N/A | N/A |
| 15. Were the results internally consistent? | N/A | N/A | N/A | N/A | N/A | N/A | D/K | N/A | N/A | N/A | N/A | N/A | D/K | Y | N/A | N/A | N/A | N/A | N/A |
| 16. Were the results/ data presented for all the analyses described in the methods/aim? | Y | N | D/K | Y | N/A | Y | Y | N/A | Y | N/A | N/A | N/A | Y | Y | N/A | Y | Y | N/A | N/A |
| 17. Were the authors' discussions and conclusions justified by the results? | Y | Y | Y | Y | Y | Y | Y | N/A | N/A | N/A | N/A | N/A | Y | Y | N/A | Y | Y | Y | N/A |
| 18 Were the limitations of the study discussed? | N | N | N | N | N/A | N | N | N/A | N | N/A | N/A | N/A | Y | Y | N/A | Y | N | N/A | N/A |
| 19. Were there any funding sources or conflicts of interest that may affect the authors’ interpretation of the results? | D/K | N | D/K | D/K | D/K | D/K | N | D/K | D/K | N/A | Y | N | N | N | N | D/K | N | N | D/K |
| 20. Was ethical approval or consent of participants attained? | D/K | N/A | N/A | D/K | N/A | N/A | D/K | N/A | N/A | N/A | N/A | N/A | Y | Y | N/A | N/A | N/A | N/A | N/A |

Table 2. AXIS definitions

| **Question** | | **Definition/Rules**  N.B. Yes/No are used where the paper explicitly addressed the relevant question. Don’t know is used when the paper does not mention the relevant question. Not Applicable is used when the paper would not need reference the relevant question. |
| --- | --- | --- |
| 1 | Were the aims/objectives of the report clear? | As per question |
| 2 | Was the study/report design appropriate for the stated aim(s)? | If responded no to question 1, automatically put Not Applicable for question 2. |
| 3 | Was the sample size justified? | Respond ‘Yes’ if a rational for sample size is included, ‘No’ if they critique the sample size anywhere in paper, and ‘Don’t know’ if sample size isn’t mentioned.  In this context, sample size refers to the number of data as per the data definition utilised in question 5.  Where the paper is a commentary or viewpoint, automatically put Not Applicable for question 3. |
| 4 | Was the target/reference population clearly defined? (Is it clear who the research was about?) | Is there a clear definition of the population/target in the abstract, introduction or method? (This does not relate to whether the results are clearly related to a set population where the population described involves multiple populations e.g. mental health healthcare workers and physical healthcare workers). |
| 5 | Was the sample frame taken from an appropriate population base so that it closely represented the target/reference population under investigation? | Was the participants/setting (e.g. mental health healthcare workers/mental health inpatient hospital) discussed representative of that population/setting in the context of the aim.  Where the paper includes anecdotal data e.g. commentary or viewpoint, automatically put Not Applicable for question 5. |
| 6 | Was the selection process likely to select subjects/participants/data that were representative of the target/reference population under investigation? | As per question.  In addition to the traditional definition of data (outcome measures, interview/focus group data), ‘data’ includes any other sources the paper has utilised to formulate/review its position. Such as literature included in literature review papers, case reviews, observation notes, hospital plans, policies, guidelines, and information gathered from Task Forces.  Commentary/viewpoints that have solely included the view/ anecdotal observation of the author are not considered data. |
| 7 | Were measures undertaken to address and categorise non-responders? | This can only be answered yes/no/don’t know where participants were recruited to take part in:   - Outcome measures - Interviews - Focus groups/Task Force - Observation notes   Literature reviews, commentaries/viewpoint and guidelines should be Not Applicable. |
| 8 | Were the outcome variables/data measured appropriate to the aims of the study? | The aforementioned definition of data is used when defining this question.  If the answer to question 1 is No, automatically put Not Applicable for question 8. |
| 9 | Were the outcome variables/data measured correctly using instruments/measurements that had been trialled, piloted or published previously? | Only the following outcome variables/data should considered as previously trialled/piloted/published data:   - Validated outcome measures - Interviews transcripts/notes - Case reviews detail - Focus groups transcripts/notes - Task Force ideas - Observation notes (where the method of obtaining these has been explicitly referenced).   Any other data should either be recorded as Don’t know, or No.  For commentaries and viewpoints, automatically put Not Applicable for question 9. |
| 10 | Is it clear what was used to determine statistical significance and/or precision estimates? (e.g. p-values, confidence intervals) | Only papers which include statistics as a way of analysis the relevant data should answer Yes/No/Don’t Know. All others should automatically put Not Applicable for question 10. |
| 11 | Were the methods (including statistical methods) sufficiently described to enable them to be repeated? | This includes papers which may not necessarily have an explicit ‘methods’ section, but still detail how data were gathered/selected in order to repeat.  For commentaries and viewpoints, automatically put Not Applicable for question 11. |
| 12 | Were the basic data adequately described? | This includes any papers that refer to data as previously defined.  For commentaries and viewpoints, automatically put Not Applicable for question 12. |
| 13 | Does the response rate raise concerns about non-response bias? | Only where the following outcome variables/data should have a Yes/No/Don’t know response for question 13:   - Validated outcome measures - Interviews transcripts/notes - Case reviews detail - Focus groups transcripts/notes - Task Force ideas - Observation notes (where the method of obtaining these has been explicitly referenced).   For all others automatically put Not Applicable for question 13. |
| 14 | If appropriate, was information about non-responders described? | As per question 13.  If responded Not Applicable to question 13, automatically put Not Applicable for question 14. |
| 15 | Were the results internally consistent? | Only papers which include statistical analysis should generate a response of Yes/No/Don’t know.  If responded Not Applicable to question 10, automatically put Not Applicable for question 15. |
| 16 | Were the results/data presented for all the analyses described in the methods/aim? | This includes any papers that refer to data as previously defined.  If responded no to question 1, automatically put Not Applicable for question 16. |
| 17 | Were the authors' discussions and conclusions justified by the results? | This is based on the reviewers assessment on whether the paper is clear regarding its data and conclusions and whether this is justified. |
| 18 | Were the limitations of the study discussed? | As per question |
| 19 | Were there any funding sources or conflicts of interest that may affect the authors’ interpretation of the results? | Response ‘Yes’ if the paper declares a conflict, ‘No’ if the paper includes a statement saying no conflict, and ‘Don’t know’ where the paper has not addressed this.  There should be no ‘Not Applicable’ responses. |
| 20 | Was ethical approval or consent of participants attained? | Yes/No/Don’t know should only be used for papers that refer to participant participation:   - Completing outcome measures - In interviews - In focus groups/task force - In observations (where the method of obtaining these has been explicitly referenced).   All other papers should automatically put Not Applicable for question 20. |
